# Dysglycaemia and Other Predictors for Progression or Regression from Impaired Fasting Glucose to Diabetes or Normoglycaemia

**DOI:** 10.1155/2015/373762

**Published:** 2015-07-27

**Authors:** L. de Abreu, Kara L. Holloway, Mark A. Kotowicz, Julie A. Pasco

**Affiliations:** ^1^School of Medicine, Deakin University, 285 Ryrie Street, Geelong, VIC 3220, Australia; ^2^Department of Medicine, NorthWest Academic Centre, The University of Melbourne, 176 Furlong Road, St Albans, VIC 3021, Australia; ^3^Barwon Health, Ryrie Street, Geelong, VIC 3220, Australia

## Abstract

*Aims*. Diabetes mellitus is a growing health problem worldwide. This study aimed to describe dysglycaemia and determine the impact of body composition and clinical and lifestyle factors on the risk of progression or regression from impaired fasting glucose (IFG) to diabetes or normoglycaemia in Australian women. *Methods*. This study included 1167 women, aged 20–94 years, enrolled in the Geelong Osteoporosis Study. Multivariable logistic regression was used to identify predictors for progression to diabetes or regression to normoglycaemia (from IFG), over 10 years of follow-up. *Results*. At baseline the proportion of women with IFG was 33.8% and 6.5% had diabetes. Those with fasting dysglycaemia had higher obesity-related factors, lower serum HDL cholesterol, and lower physical activity. Over a decade, the incidence of progression from IFG to diabetes was 18.1 per 1,000 person-years (95% CI, 10.7–28.2). Fasting plasma glucose and serum triglycerides were important factors in both progression to diabetes and regression to normoglycaemia. *Conclusions*. Our results show a transitional process; those with IFG had risk factors intermediate to normoglycaemics and those with diabetes. This investigation may help target interventions to those with IFG at high risk of progression to diabetes and thereby prevent cases of diabetes.

## 1. Introduction

Diabetes mellitus is a complex and often silent chronic disease that can result in serious consequences, potentially leading to premature mortality [1]. Diabetes is a serious health problem; it impacts the lives of approximately 347 million people worldwide [2] and in Australia, at present, over 1.5 million people have diabetes. It is the fastest growing chronic disease in Australia, with 280 people newly diagnosed every day [3]. Impaired fasting glucose (IFG) is defined as a blood glucose level higher than “normal” but lower than the threshold for diagnosis of diabetes. Since 2003, the American Diabetes Association (ADA) has classified IFG as fasting plasma glucose (FPG) level between 5.5 and 6.9 mmol/L (100 to 125 mg/dL), without antihyperglycaemic medication [4]. It is known that IFG is a risk factor for diabetes and cardiovascular disease [5–10], but few studies have shown the epidemiology of progression from IFG to diabetes in the Australian population.

Research shows that a number of anthropometric and metabolic factors can influence the risk of developing diabetes. These include overweight (body mass index; BMI > 25 kg/m^2^) and obesity (BMI > 30 kg/m^2^), which are associated with higher risk of developing diabetes compared to normal weight [11, 12]. An increased waist circumference has also been shown to increase the risk of developing diabetes [12]. The addition of all other related factors, including hypertension, elevated serum triglycerides (≥1.7 mmol/L), lowered HDL cholesterol (<1.29 mmol/L), and IFG (≥5.6 mmol/L), independently increases the risk of developing diabetes in an additive way [12]. In Australia, the prevalence of these factors is high; 39.1% of adults are overweight and 20.5% are obese [13]. A further 20.5% had elevated triglycerides and 28.8% have hypertension. Lifestyle related factors including smoking and physical inactivity are also high, 15.6% and 50.2%, respectively. Given these high levels of factors that have been shown to increase the risk of developing diabetes, the aim of this study was to describe dysglycaemia, evaluate the progression of isolated IFG to diabetes or its regression to normoglycaemia in Australian women, and understand how body composition and clinical and lifestyle factors affect these changes.

## 2. Patients and Methods

### 2.1. Study Design and Subjects

This study utilises data from the female arm of the Geelong Osteoporosis Study (GOS), a population-based study established in the Barwon Statistical Division (BSD). The BSD is located in south-eastern Australia and has a large, stable population of approximately 250,000 with an extended range of cultural and socioeconomic characteristics. The BSD is representative of the Australian population, making it ideal for epidemiological studies. A complete description of the methodology has been published elsewhere [14]. At baseline, 1993–1997, an age-stratified random sample of 1494 women aged 20–94 years was recruited from Commonwealth electoral rolls with a participation of 77.1%. For this analysis, we excluded 326 women because we did not have a FPG level or self-report of antihyperglycaemic medication or diabetes status. Thus, 1167 women were eligible for baseline analysis. In the second phase of this analysis, we focused on 187 women who had IFG at baseline and whose diabetes status was reassessed a decade later, 2004–2008.

The study was approved by the Barwon Health Human Research Ethics Committee, and written, informed consent was obtained from all subjects.

### 2.2. Measurements

At baseline, weight and height were measured to the nearest ±0.1 kg and ±0.1 cm, respectively, and body mass index (BMI) was calculated as weight/height^2^ (kg/m^2^). Subjects were considered obese if BMI ≥ 30.0 kg/m^2^ [15]. Waist circumference was measured to the nearest ±0.5 cm at the minimum circumference between the lowest ribs and the iliac crest and was defined as normal if it was <80.0 cm [16]. Hip circumference (cm) was measured at the maximal gluteal position; the waist-to-hip ratio (WHR) and waist-to-height ratio (WHtR) were calculated and defined as normal if their values were <0.80 [17] and <0.5 [18], respectively. Whole body scans were performed using a dual-energy X-ray absorptiometry (DXA; Lunar DPX-L; Lunar, Madison, WI), which provided estimates of body fat mass (kg) and “lean” mass (kg), comprising muscle, skin, connective tissue, and the lean component of adipose tissue—water and protein [19]. Blood pressure (seated) was measured using an automated meter (Takeda Medical UA-751). Hypertension was defined as a systolic pressure ≥140 mmHg and/or a diastolic pressure ≥90 mmHg and/or use of antihypertensive medication [16]. Physical activity, alcohol consumption, current smoking, and medication use were determined by questionnaire sent out to the participants. Physical activity was collected from a multiple choice question with the possible responses being “very active, active, sedentary, limited, inactive, chair/bedridden, and bedfast.” These responses were categorised; “very active” and “active” were pooled as “high” physical activity and the other categories were collapsed and defined as “low.” Alcohol consumption was collected in a similar way, with the responses including “never, less than once per week, once or twice per week, several times per week, and every day.” “High” alcohol consumption was defined as those who consumed alcoholic beverages “several times per week” or “every day.” All other responses for this question were classified as “low” alcohol consumption. Women who reported undertaking regular physical activity were described as active; otherwise they were classified as inactive; high alcohol consumption was recognised if alcohol was consumed at least several times a week; antihyperglycaemic medication use referred to medications taken regularly and currently at baseline.

Venous blood was collected after an overnight fast at both baseline and 10-year follow-up; plasma glucose levels were measured together with serum levels of triglycerides, high density lipoprotein (HDL) cholesterol, and low density lipoprotein (LDL) cholesterol using standard laboratory methods. The method for measuring fasting glucose was an adaptation of the hexokinase-glucose-6-phosphate dehydrogenase method [20]. Total cholesterol, high density lipoprotein cholesterol (HDL-C), low density lipoprotein cholesterol (LDL-C), and triglycerides were determined using commercially available kits (Thermo Fisher Scientific), with analysis completed on a CDX90 automated clinical chemistry analyser (Thermo Fisher Scientific), following manufacturer's instructions.

We also examined the use of lipid lowering medications, but few women used these agents (*n* = 51), and among those who did, serum lipid results were still outside the recommended “target range.” Diabetes was by having FPG ≥7.0 mmol/L [4] and/or by the self-reported diabetes and/or by the use of antihyperglycaemic agents [11]. We defined IFG according to the 2003 ADA diagnostic criteria, 5.5–6.9 mmol/L [4]. We also determined whether the participants had metabolic syndrome according to the International Diabetes Federation (IDF) criteria (2005 revision [21]), which included measurements of waist circumference, FPG, serum triglycerides, serum HDL, and hypertension. Briefly, if a participant had a waist circumference >80 cm and at least two of the following: (i) raised TG level: ≥1.7 mmol/L, or specific treatment for this lipid abnormality; (ii) reduced HDL cholesterol: <1.29 mmol/L, or specific treatment for this lipid abnormality; (iii) raised blood pressure: systolic BP ≥ 130 or diastolic BP ≥ 85 mmHg, or treatment of previously diagnosed hypertension; (iv) raised FPG ≥ 5.6 mmol/L, or previously diagnosed type 2 diabetes, then the participant was considered to have metabolic syndrome. In addition, since we analysed the data in groups based on normoglycaemia, IFG, and diabetes, we also examined all of the metabolic syndrome criteria while excluding FPG.

### 2.3. Statistical Analysis

Differences in subject characteristics were identified using Kruskal-Wallis test for continuous data according to the three glycaemic categories (normal, IFG, and diabetes) at both baseline and 10-year follow-up. The Chi-square test (or Fisher's exact test) was used to determine differences between data when expressed in discrete categories. Multivariable logistic regression models were used to identify risk factors for progressing from IFG at baseline to diabetes over the 10 years of follow-up as well as predictors for regression to normoglycaemia over the same time period. Odds ratios for potential risk factors were calculated. Lipid profiles were missing for six participants, so statistical modelling was performed using *n* = 181. The following factors were included in the logistic model as continuous variables measured at baseline: age (years), BMI (kg/m^2^), waist circumference (cm), hip circumference (cm), body fat mass (kg), lean mass (kg), serum HDL cholesterol (mmol/L), and serum LDL cholesterol (mmol/L). Additional factors were included into the same model as categorical variables: fasting glucose at baseline (above or below 6.1 mmol/L), serum triglycerides (above or below 1.7 mmol/L), hypertension (yes/no), current smoking (yes/no), high alcohol consumption (yes/no), physical activity (high/low), and metabolic syndrome (yes/no). Variables to be tested in the final model were identified using univariate analysis and those with *p* < 0.05 were selected for inclusion. Variables that contributed to the model by altering the point estimate for the odds ratio and retained *p* < 0.05 were included in the final model. All logistic models were adjusted for age. Variables in the final model were tested for interaction. Statistical analyses were conducted using the MINITAB software package (Version 16; Minitab, State College, PA, USA).

### 2.4. Prevalence and Incidence Rate Calculations

Data from the Australian Bureau of Statistics 1996 Census Community Profile Series for the Australian Population (catalogue number: 2020.0) were used to calculate age-standardised prevalence of IFG and diabetes at baseline. The age-standardised incidence of new diabetes cases from those who had progressed from IFG was calculated over a 10-year period, using data from the Australian Bureau of Statistics 2006 Census Community Profile Series for the Australian Population (catalogue number 2001.0).

## 3. Results

### 3.1. Cross-Sectional Baseline Data 

**Figure 1:**
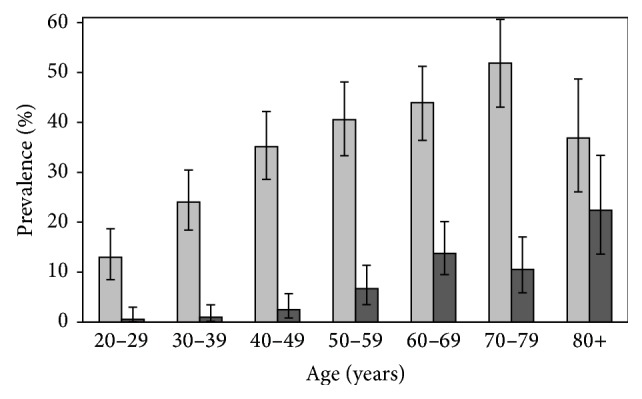
Mean age-specific prevalence of diabetes mellitus and impaired fasting glucose (IFG) for women at baseline. Error bars represent 95% CIs.

Subject characteristics at baseline are shown in Table 1. Among 1167 women, 696 (59.6%) had normoglycaemia, 395 (33.8%) had IFG, and 76 (6.5%) met criteria for diabetes. There was a pattern of increasing median age across the normoglycaemic, IFG, and diabetes groups. There was an age-related increase in the prevalence of IFG, ranging from approximately 13% for the age of 20–29 years and peaking at approximately 50% for the age of 70–79 years (Figure 1). A similar age-related increase was observed for diabetes, however, at lower prevalence, rising from 0.5% for the age of 20–29 and peaking at 22.4% for those aged 80 years and older. Age-standardised prevalence of IFG and diabetes was 31.5% (95% CI, 28.4–34.5) and 5.6% (95% CI, 4.5–6.7), respectively, for the ages of 20 years and older.

There was a consistent pattern of increasing weight, BMI, waist circumference, hip circumference, body fat mass, WHR, WHtR, serum triglycerides, and systolic and diastolic blood pressure in the groups with dysglycaemia (IFG and diabetes). Similarly, there was a pattern of increasing obesity and low physical activity in those with IFG and diabetes. There was an inverse pattern observed with serum HDL cholesterol levels; lower levels were observed with increasing severity of dysglycaemia. More than 60% of women at baseline with IFG or diabetes had metabolic syndrome, whereas only approximately 20% of those with normoglycaemia were affected. When considering metabolic syndrome without FPG, the results were different. There was a gradual increase across the groups, with approximately 20% in the normal plasma glucose group, 39% in the IFG group, and 68% of those with diabetes.

The women who were excluded from the study due to insufficient information to classify diabetes status differed from those who were included in the study. Those who were excluded were older and had lower weight, shorter height, lower lean mass, greater waist circumference, higher systolic and diastolic blood pressure, higher serum triglycerides, and lower serum HDL cholesterol, with a lower proportion of smokers and lower mobility.

### 3.2. Progression from IFG to Diabetes over Follow-Up

**Figure 2:**
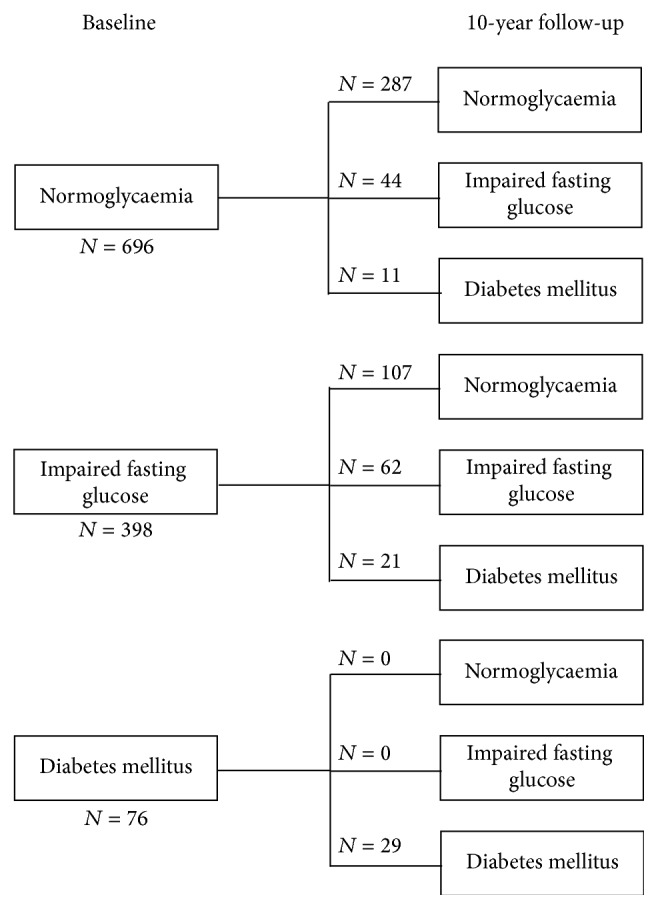
Numbers of women in each of the three glycaemia groups who became normoglycaemic and developed impaired fasting glucose or diabetes over the 10-year follow-up. Note: missing data for 354 women with normoglycaemia at baseline, 208 women with impaired fasting glucose at baseline, and 47 women with diabetes at baseline.

All women with diabetes at baseline were again classified as diabetics at the 10-year follow-up (Figure 2). Among 335 women with normoglycaemia at baseline, 280 (83.6%) remained in this category at 10-year follow-up, 44 (13.1%) changed to IFG, and 11 (3.3%) progressed to diabetes. Among 187 women with IFG at baseline, 62 (33.2%) remained IFG, 104 (55.6%) reverted to normoglycaemia, and 21 (11.2%) developed diabetes. Characteristics of those with IFG at baseline are shown in Table 2, together with a comparison of characteristics for those who remained in the IFG group, those who progressed to diabetes, and those who regressed to normoglycaemia. A comparison between the three groups showed that those who progressed to diabetes had higher FPG and greater indices of adiposity including weight, BMI, body fat mass, waist and hip circumference, WHR, WHtR, systolic blood pressure, hypertension, serum triglycerides, and obesity; they also had greater lean body mass and lower serum HDL. When analysing metabolic syndrome, the results were similar to Table 1; more than 70% of those who remained in the IFG group or progressed to diabetes were affected. Only about half of those who regressed to normoglycaemia had metabolic syndrome. The results for metabolic syndrome when FPG was excluded showed a different pattern; in all glycaemia groups, fewer women were classified as having metabolic syndrome. There was a gradual increase in proportions of those with metabolic syndrome across the groups, from 25.0% in the normoglycaemic group to 46.8% in the IFG group and 61.9% in the diabetic group.

### 3.3. Incidence of Progression from IFG to Diabetes over a Decade

During 1768 person-years of follow-up, 21 of 187 women with IFG at baseline progressed to diabetes. This corresponded to an age-standardised incidence rate of 18.1 per 1,000 person-years (95% CI, 10.7–28.2).

### 3.4. Risk Factors for IFG to Diabetes

In a multivariable model including 181 participants, FPG and serum triglycerides were identified as independent risk factors for progressing from IFG to diabetes over the ensuing decade (Table 3). The odds ratio for progressing to diabetes was nearly sixfold greater if FPG ≥6.1 mmol/L (OR 5.75 (95% CI, 1.86–17.78), *p* = 0.002) and nearly eightfold greater if serum triglycerides ≥1.7 mmol/L (OR 7.86 (95% CI, 2.76–22.38), *p* < 0.001). These relationships were not explained by differences in age, body fatness, blood pressure, health behaviours, serum HDL cholesterol levels, or serum LDL cholesterol levels. We also examined metabolic syndrome as a predictor for progression from IFG to diabetes, but it was not significant (OR 1.85, 95% CI, 0.59–5.84).

### 3.5. Predictors for Regression from IFG to Normoglycaemia

A multivariable model also showed that FPG, lean mass, and serum triglycerides were independent predictors of regression from IFG to normoglycaemia (Table 3). The odds ratio for FPG was 0.19 (95% CI, 0.05–0.70, *p* = 0.012), meaning that those with higher FPG had a reduced likelihood of regressing to normoglycaemia over the 10 years of follow-up. Serum triglycerides and lean mass followed similar patterns, with odds ratios of 0.46 (0.26–0.81, *p* = 0.008) and 0.87 (95% CI, 0.80–0.94, *p* = 0.001), respectively. No interaction terms were identified. In addition, we also analysed absence of metabolic syndrome as a predictor of regression to normoglycaemia; however, the results were not significant (OR 0.61, 95% CI, 0.28–1.34).

## 4. Discussion

This longitudinal and cross-sectional population-based study reports on progression to diabetes and regression to normoglycaemia from IFG in a female Australian cohort over a 10-year period. Individuals with IFG or diabetes at baseline had older age, greater indices of adiposity, higher blood pressure, serum triglycerides, serum LDL cholesterol, alcohol consumption, and more physical inactivity. High FPG and high serum triglycerides were identified as independent predictors for the progression from IFG to diabetes, while lower levels of FPG and serum triglycerides were independent predictors of regression to normoglycaemia. Lean mass was also an independent predictor for the regression from IFG to normoglycaemia. We also analysed metabolic syndrome as a risk factor, but it was not significant in statistical analyses. Indeed, it has been shown that metabolic syndrome may not be more effective than the individual components that are included in its calculation [22], which may explain why we did not observe it as a predictive factor for diabetes classifications.

There have been many studies that report on the incidence of IFG and risk factors for progression to diabetes, which are collated in a meta-analysis by Morris et al. [23]. However, many of them did not use the ADA criteria and consequently are not directly comparable to our study. One study, which did use the ADA criteria [8], investigated a cohort of European men and women and reported similar results to our study for IFG (compared to normoglycaemia) including increased age, BMI, waist circumference, serum HDL cholesterol, serum triglycerides, and blood pressure. They also reported an incidence for progression from IFG to diabetes of 10.6 per 1,000 person-years (95% CI: 8.1–13.9), similar in magnitude to our value. Differences between our study and the Forouhi study include that the latter considered adults aged between 40 and 69 years, whereas we investigated the entire adult age range (20–94 years). Additionally, their study included a higher proportion of adults living in more affluent areas whereas our study included adults from range of socioeconomic levels [19].

There is also a prospective study from the USA which reported diabetes incidence using data from men and women aged 30 years or older [24]. In this study 17.1% of those with IFG developed diabetes within five years, corresponding to an incidence rate of 37.4 per 1,000 person-years (95% CI, 36.0–38.9). Differences between study subjects, such as the inclusion of men and women, sample size, levels of adiposity, life style/environment, and ethnicity, may account for differences between our results and the results of this study.

Other studies have examined risk factors for the progression of IFG to diabetes. One such study by Bonora et al. [11] reported that age, BMI, IFG, and impaired glucose tolerance (IGT) were all predictors of diabetes. Aekplakorn et al. [25] developed a model of risk factors including age, BMI, waist circumference, hypertension, and history of diabetes (parents/siblings), with only a small increase in quality of their model when IFG, IGT, serum HDL cholesterol, and/or triglycerides were included. Another investigation in Chinese participants showed that, after adjustment for age, sex, smoking, alcohol consumption, and a family history of hypertension, obesity, diabetes, and hyperlipidaemia, only IFG was an independent risk factor for diabetes [26]. The results from our analysis are similar, showing that IFG (FPG levels) and serum triglycerides were independent predictors of progression from IFG to diabetes. We did not observe an independent association between diabetes and age, BMI, WC, WHR, or hypertension; however, individuals with IFG were more likely to be overweight and consequently have higher BMI, waist circumference, and so forth (see Table 2). Therefore, IFG alone may act as a surrogate for fat mass and obesity in this cohort.

There have been studies investigating the regression from IFG to normoglycaemia, almost always using medication rather than modifiable lifestyle factors [27]. In our study, none of the participants classified with IFG (by definition) were taking any antihyperglycaemic agents that would influence their regression to normoglycaemia. Our data showed that relatively low FPG, serum triglycerides, and lean mass were predictors for regression to normoglycaemia and in addition, measures of adiposity were lower in those who regressed to normoglycaemia. These predictors are associated with improved health, except for higher lean mass, which was associated with nonregression. In order to investigate this, we calculated a ratio of lean/total weight for IFG subjects who either regressed to normoglycaemia or remained in the IFG group at the 10-year follow-up of the study (data not shown). Those who regressed to normoglycaemia had a higher lean/total weight ratio (0.56) than those who remained in the IFG group (0.53) (*p* = 0.006) (data not shown). This indicates that those who remained in the IFG group had higher lean mass, but it contributed a lower proportion of total body weight than those who regressed to normoglycaemia.

Our study has some strengths and limitations. The major strengths are that the participants were randomly selected, which is important when estimating the age-standardised prevalence of diabetes in the region. Our study also included a wide age range with considerable follow-up time. We also used a robust method for the diagnosis of diabetes, which included a FPG measurement, self-report, and medication use. The study also utilised whole body densitometry for the assessment of body fat mass, lean mass, and BMI more accurately than typical anthropometric measurements. However, we acknowledge that there are some limitations to the present study. Most of the participants were white females and our results may not be generalisable to other populations. The women who were excluded from the study due to insufficient information to classify diabetes status differed from those who were included in the study. Those who were excluded were older and had lower weight, shorter height, lower lean mass, greater waist circumference, higher systolic and diastolic blood pressure, higher serum triglycerides, lower serum HDL cholesterol, with a lower proportion of smokers and lower mobility. We acknowledge that this is a limitation in determining the incidence of progression to diabetes in this study and our results are possibly a conservative estimate. However, our estimate is comparable to others reported in the literature. Additionally, there was some attrition during the course of the study and there may have been differential loss to follow-up; it was not possible to determine if those lost to follow-up were more or less likely to have developed diabetes than those who were retained in the study. Harmonization of the IDF and ADA criteria has resulted in a small increase in the prevalence of the metabolic syndrome in some populations. Given that the impact of the metabolic syndrome on the likelihood of progressing to diabetes was far from significant (Supplementary Table available online at http://dx.doi.org/10.1155/2015/373762), using different criteria for defining the metabolic syndrome is unlikely to impact our reported findings. Finally, we did rely on some self-reported data such as medication use, smoking, alcohol consumption, and physical activity, which may not be accurate, but it is important to note that most of our analyses were based on biochemical and clinical measurements.

## 5. Conclusions

In conclusion, we achieved the study objective, to describe the epidemiology of dysglycaemia and the impact of body composition and clinical and lifestyle factors on the risk of progression to diabetes and regression to normoglycaemia in a cohort of Australian women. We report that women with dysglycaemia had higher obesity-related factors and lower serum HDL cholesterol. There was an age-related increase in the prevalence of IFG and diabetes. FPG and serum triglycerides were revealed as independent predictors of progression from IFG to diabetes. Independent predictors for regression from IFG to normoglycaemia included FPG, serum triglycerides, and lean mass.

## Supplementary Material

The Supplementary Table shows the non-significant results of the logistic analyses for all factors investigated in this study. The odds ratios are presented for both progression from impaired fasting glucose (IFG) to diabetes and regression to normoglycaemia over the 10 year follow-up period.

## Figures and Tables

**Table 1:** Subject characteristics according to diabetes status at baseline (normal fasting glucose (NFG), impaired fasting glucose (IFG), and diabetes). Data are shown as median (interquartile range) or *n* (%).

	All (*n* = 1167)	NFG (*n* = 696)	IFG (*n* = 395)	Diabetes (*n* = 76)	*p*
Fasting plasma glucose (mmol/L)^*∗*^	5.3 (5.0–5.7)	5.0 (4.8–5.2)	5.7 (5.6–5.9)	8.1 (6.6–11.1)	<0.001
Age (years)	49.3 (35.2–65.0)	42.1 (31.1–42.1)	56.8 (44.0–64.4)	65.2 (59.8–75.3)	<0.001
Weight (kg)	66.3 (59.1–76.9)	63.8 (57.3–72.8)	69.5 (61.1–80.8)	71.5 (60.8–83.2)	<0.001
BMI (kg/m^2^)	25.4 (22.6–29.5)	24.5 (22.0–27.7)	26.9 (23.9–31.4)	30.0 (25.6–33.3)	<0.001
Body fat mass (kg)^*∗*^	25.3 (19.3–32.8)	23.0 (17.5–30.3)	28.0 (22.1–36.2)	29.5 (21.0–37.1)	<0.001
Lean mass (kg)^*∗*^	38.5 (35.7–41.6)	38.4 (35.9–41.3)	38.6 (35.4–42.2)	38.7 (36.1–41.9)	0.69
Waist circumference (cm)^*∗*^	82.6 (74.6–92.7)	79.0 (72.5–87.4)	87.0 (78.6–96.5)	96.4 (87.1–104.6)	<0.001
Hip circumference (cm)^*∗*^	103.3 (97.1–111.4)	101.2 (96.2–108.3)	106.3 (99.3–106.3)	108.7 (98.9–119.8)	<0.001
WHR (waist-to-hip ratio)^*∗*^	0.80 (0.75–0.84)	0.78 (0.74–0.83)	0.82 (0.77–0.86)	0.88 (0.84–0.91)	<0.001
WHtR (waist-to-height ratio)^*∗*^	0.51 (0.46–0.58)	0.49 (0.44–0.55)	0.54 (0.49–0.60)	0.62 (0.56–0.67)	<0.001
Systolic blood pressure (mmHg)^*∗*^	121.0 (108.0–136.0)	114.0 (104.0–128.0)	128.0 (115.0–140.0)	141.0 (126.3–163.5)	<0.001
Diastolic blood pressure (mmHg)^*∗*^	76.0 (68.0–84.0)	74.0 (66.0–82.0)	79.0 (71.0–86.0)	78.5 (73.0–92.8)	<0.001
Serum triglycerides (mmol/L)^*∗*^	1.08 (0.75–1.58)	0.94 (0.69–1.36)	1.26 (0.89–1.71)	2.01 (1.38–2.42)	<0.001
Serum HDL cholesterol (mmol/L)^*∗*^	1.21 (0.98–1.47)	1.23 (1.02–1.49)	1.20 (0.95–1.45)	0.98 (0.84–1.26)	<0.001
Serum LDL cholesterol (mmol/L)^*∗*^	2.88 (2.30–3.53)	2.71 (2.20–3.36)	3.13 (2.55–3.92)	2.96 (2.23–3.44)	<0.001
Obesity (%)	266.0 (22.8)	104.0 (14.9)	124.0 (31.4)	38.0 (49.4)	<0.001
Hypertension (mmHg) (%)^*∗*^	427 (36.6)	184 (26.4)	181 (45.8)	62 (81.6)	<0.001
Current smoker (%)	189 (16.2)	120 (17.1)	56 (14.2)	13 (17.1)	0.40
High alcohol consumption (%)	210 (18.0)	115 (16.5)	89 (22.5)	6 (7.9)	0.003
Low physical activity (%)	320 (27.4)	146 (21.0)	127 (32.2)	47 (61.8)	<0.001
Metabolic syndrome (%)	459 (39.3)	150 (21.6)	252 (63.8)	57 (75.0)	<0.001
“Other” metabolic syndromes^†^	356 (30.5)	150 (21.6)	154 (39.0)	2 (68.4)	<0.001

^*∗*^Missing data: fasting plasma glucose and blood pressure *n* = 22; body fat and lean mass *n* = 8; waist, hip circumference, waist-to-hip ratio, and waist-to-height ratio *n* = 13; serum triglycerides *n* = 63; serum HDL cholesterol *n* = 61; serum LDL cholesterol *n* = 59.

^†^Metabolic syndrome excluding FPG.

**Table 2:** Characteristics of subjects with impaired fasting glucose (IFG) at baseline showing those who progressed to diabetes mellitus and those who regressed to normal fasting glucose (NFG) over the decade of follow-up. Data are shown as median (interquartile range) or *n* (%).

	All (*n* = 187)	NFG (*n* = 104)	IFG (*n* = 62)	Diabetes (*n* = 21)	*p*
Fasting plasma glucose (mmol/L)^*∗*^	5.3 (5.0–5.8)	5.7 (5.5–5.8)	5.7 (5.6–6.0)	5.9 (5.7–6.4)	<0.001
Age (years)	53.8 (44.0–64.4)	52.4 (41.5–65.0)	55.5 (45.8–64.2)	53.4 (43.7–65.9)	0.73
Weight (kg)	71.3 (62.7–81.1)	67.0 (60.5–76.3)	73.8 (67.1–86.1)	79.9 (74.3–96.0)	<0.001
BMI (kg/m^2^)	27.7 (24.3–31.4)	25.9 (23.8–29.3)	28.8 (25.0–32.6)	30.5 (26.7–35.6)	<0.001
Body fat mass (kg)	28.5 (22.8–36.4)	26.7 (21.4–32.8)	30.8 (25.5–38.5)	35.2 (27.8–44.8)	<0.001
Lean mass (kg)	39.2 (35.9–42.4)	37.6 (35.1–41.1)	40.5 (37.4–43.6)	42.4 (38.6–45.4)	<0.001
Waist circumference (cm)	86.3 (78.0–94.2)	84.3 (74.5–90.3)	90.6 (82.4–101.2)	97.1 (87.0–104.6)	<0.001
Hip circumference (cm)	106.3 (100.0–114.6)	103.9 (99.1–109.8)	111.1 (102.7–118.7)	111.9 (104.9–125.6)	<0.001
WHR (waist-to-hip ratio)	0.81 (0.77–0.86)	0.79 (0.75–0.85)	0.83 (0.77–0.88)	0.84 (0.81–0.88)	0.004
WHtR (waist-to-height ratio)	0.54 (0.48–0.59)	0.52 (0.47–0.57)	0.56 (0.51–0.62)	0.58 (0.53–0.64)	<0.001
Systolic blood pressure (mmHg)^*∗*^	127.5 (114.3–139.8)	121.0 (111.0–135.0)	133.0 (123.0–141.5)	132.0 (120.5–141.0)	0.004
Diastolic blood pressure (mmHg)^*∗*^	79.0 (72.0–86.0)	77.0 (71.0–84.0)	81.0 (73.0–89.8)	80.0 (73.5–88.0)	0.18
Serum triglycerides (mmol/L)^*∗*^	1.13 (0.80–1.61)	1.02 (0.76–1.37)	1.25 (0.88–1.63)	1.84 (1.02–2.88)	<0.001
Serum HDL cholesterol (mmol/L)^*∗*^	1.21 (0.95–1.45)	1.26 (1.04–1.57)	1.15 (0.89–1.41)	0.93 (0.83–1.21)	0.002
Serum LDL cholesterol (mmol/L)^*∗*^	3.06 (2.41–3.71)	3.02 (2.35–3.53)	3.00 (2.55–4.03)	3.33 (2.63–3.90)	0.24
Obesity (%)	62 (33.2)	24 (23.1)	27 (43.6)	11 (52.4)	0.004
Hypertension^*∗*^	80 (42.8)	34 (32.7)	34 (54.8)	12 (57.1)	0.008
Current smoker	25 (13.4)	12 (11.5)	10 (16.1)	3 (14.3)	0.7
High alcohol consumption	44 (23.5)	26 (25.0)	15 (24.2)	3 (14.3)	0.57
Low physical activity	44 (23.5)	21 (20.2)	19 (30.7)	4 (19.1)	0.27
Metabolic syndrome (%)	112 (59.9)	51 (49.0)	46 (74.2)	15 (71.4)	0.003
“Other” metabolic syndromes^†^	68 (36.4)	26 (25.0)	29 (46.8)	13 (61.9)	<0.001

^*∗*^Missing data: serum fasting plasma glucose *n* = 4; blood pressure *n* = 3; serum triglycerides and serum HDL cholesterol *n* = 6; serum LDL cholesterol *n* = 5.

^†^Metabolic syndrome excluding FPG.

**Table 3:** Odds ratios for independent predictors from the logistic regression for progression from impaired fasting glucose (IFG) at baseline to diabetes or regression to normoglycaemia over a 10-year follow-up period in women. Of 181 women, 21 developed diabetes over the follow-up period and 104 reverted to normoglycaemia. Data are presented as OR (95% CI).

	Progression to diabetes	*p* value	Regression to normoglycaemia	*p* value
Fasting plasma glucose ≥6.1 mmol/L	5.75 (1.86, 17.78)	0.002	0.19 (0.05, 0.70)	0.012
Serum triglycerides ≥1.7 mmol/L	7.86 (2.76, 22.38)	<0.001	0.46 (0.26, 0.81)	0.008
Lean mass (kg)	NS	NS	0.87 (0.80, 0.94)	0.001

NS = not significant.
