# Supplementary material for: Dysglycaemia and Other Predictors for Progression or Regression from Impaired Fasting Glucose to Diabetes or Normoglycaemia
Source: J Diabetes Res. 2015 Jul 27;2015:373762. doi: 10.1155/2015/373762 (PMC4530268; doi:10.1155/2015/373762)
Supplement: Supplementary file 1 — The Supplementary Table shows the non-significant results of the logistic analyses for all factors investigated in this study. The odds ratios are presented for both progression from impaired fasting glucose (IFG) to diabetes and regression to normoglycaemia over the 10 year follow-up period. [file 373762.f1.docx]

Supplementary Table: Non-significant odds ratios for proposed predictors from the logistic regression for progression from impaired fasting glucose (IFG) at baseline to diabetes or regression to normoglycaemia over a 10 year follow-up period in women. Of 181 women, 21 developed diabetes over the follow up period and 104 reverted to normoglycaemia. Data presented OR (95%CI). The results are stratified by univariate and multivariate analyses since some variables that were significant in the univariate analyses were not significant in the multivariate analysis.

|  | Progression to diabetes | | | | Regression to normoglycaemia | | | |
| --- | --- | --- | --- | --- | --- | --- | --- | --- |
|  | Univariate model | P value | Multivariate model | P value | Univariate model | P value | Multivariate model | P value |
| Age (years) | 1.01 (0.97, 1.04) | 0.688 | - | - | 0.99 (0.97, 1.01) | 0.379 | - | - |
| BMI (kg/m^2^) | 1.10 (1.02, 1.18) | 0.012 | 1.02 (0.76, 1.37) | 0.894 | 0.87 (0.82, 0.93) | <0.001 | 1.01 (0.82, 1.25) | 0.932 |
| Waist circumference (cm) | 1.05 (1.02, 1.09) | 0.004 | 1.02 (0.98, 1.07) | 0.299 | 0.94 (0.91, 0.96) | <0.001 | 0.97 (0.92, 1.02) | 0.259 |
| Hip circumference (cm) | 1.04 (1.01, 1.08) | 0.019 | 0.97 (0.89, 1.07) | 0.581 | 0.94 (0.91, 0.97) | <0.001 | 0.97 (0.92, 1.03) | 0.382 |
| Body fat mass (kg) | 1.05 (1.01, 1.10) | 0.018 | 1.04 (0.90, 1.20) | 0.600 | 0.94 (0.91, 0.97) | <0.001 | 1.06 (0.97, 1.17) | 0.199 |
| Lean mass (kg)* | 1.13 (1.03, 1.23) | 0.011 | 1.01 (0.81, 1.24) | 0.961 | - | - | - | - |
| Serum HDL cholesterol (mmol/L) | 0.15 (0.03, 0.73) | 0.018 | 1.08 (0.20, 5.82) | 0.930 | 4.00 (1.69, 9.49) | 0.002 | 1.68 (0.60, 4.71) | 0.322 |
| Serum LDL cholesterol (mmol/L) | 1.32 (0.80, 2.15) | 0.276 | - | - | 0.76 (0.55, 1.05) | 0.099 | - | - |
| Hypertension (yes/no) | 1.92 (0.77, 4.81) | 0.163 | - | - | 0.39 (0.22, 0.71) | 0.002 | 0.61 (0.29, 1.29) | 0.197 |
| Smoking (yes/no) | 1.09 (0.30, 4.01) | 0.896 | - | - | 0.70 (0.30, 1.63) | 0.412 | - | - |
| High alcohol consumption (yes/no) | 0.51 (0.14, 1.81) | 0.297 | - | - | 1.20 (0.61, 2.39) | 0.596 | - | - |
| Physical activity (high/low) | 0.74 (0.24, 2.33) | 0.608 | - | - | 0.66 (0.33, 1.30) | 0.230 | - | - |
| Metabolic syndrome (yes/no) | 1.78 (0.66, 4.81) | 0.257 | - | - | 0.35 (0.19, 0.65) | 0.001 | 1.31 (0.49, 3.47) | 0.591 |

* Note that in the analysis for regression from impaired fasting glucose to normoglycaemia, lean mass was significant in the final model.
